# TLR4 signaling induces TLR3 up-regulation in alveolar macrophages during acute lung injury

**DOI:** 10.1038/srep34278

**Published:** 2017-02-15

**Authors:** Xibing Ding, Shuqing Jin, Yao Tong, Xi Jiang, Zhixia Chen, Shuya Mei, Liming Zhang, Timothy R. Billiar, Quan Li

**Affiliations:** 1 Department of Anesthesiology, East Hospital, Tongji University School of Medicine, Shanghai, China; 2 Department of Anesthesiology, University of Pittsburgh School of Medicine, Pittsburgh, PA, USA; 3 Department of Surgery, University of Pittsburgh School of Medicine, 200 Lothrop St, Pittsburgh, PA 15213, USA

## Abstract

Acute lung injury is a life-threatening inflammatory response caused by severe infection. Toll-like receptors in alveolar macrophages (AMΦ) recognize the molecular constituents of pathogens and activate the host’s innate immune responses. Numerous studies have documented the importance of TLR-TLR cross talk, but few studies have specifically addressed the relationship between TLR4 and TLR3. We explored a novel mechanism of TLR3 up-regulation that is induced by LPS-TLR4 signaling in a dose- and time-dependent manner in AMΦ from C57BL/6 mice, while the LPS-induced TLR3 expression was significantly reduced in TLR4^−/−^ and Myd88^−/−^ mice and following pretreatment with a NF-κB inhibitor. The enhanced TLR3 up-regulation in AMΦ augmented the expression of cytokines and chemokines in response to sequential challenges with LPS and Poly I:C, a TLR3 ligand, which was physiologically associated with amplified AMΦ-induced PMN migration into lung alveoli. Our study demonstrates that the synergistic effect between TLR4 and TLR3 in macrophages is an important determinant in acute lung injury and, more importantly, that TLR3 up-regulation is dependent on TLR4-MyD88-NF-κB signaling. These results raise the possibility that bacterial infections can induce sensitivity to viral infections, which may have important implications for the therapeutic manipulation of the innate immune system.

Acute lung injury (ALI) and the more severe form termed acute respiratory distress syndrome (ARDS) are associated with an estimated mortality of 40–50%^1^. Causes of ALI may be direct (pneumonia, aspiration, inhalational injury, etc.) or indirect (sepsis, pancreatitis, blood transfusion, etc.). ALI is characterized by increased vascular permeability caused by dysfunction of the alveolar-capillary membrane, lung edema, neutrophil-derived inflammation, and surfactant dysfunction^2^. During the course of ALI/ARDS, resident lung cells such as alveolar macrophages (AMΦ) are stimulated to release chemoattractants, which recruit inflammatory cells to migrate from the intravascular space across the endothelium and epithelium into the airspaces^3^.

Macrophage activation in response to microbial infection depends on Toll-like receptors (TLRs), which are a family of pattern recognition receptors (PRRs) and key regulators of both innate and adaptive immunity^4^. TLRs are among the most well-studied PRRs because of their ability to detect a variety of pathogen-associated molecular patterns (PAMPs), such as lipids, proteins, lipoproteins, and nucleic acids^5^,^6^. To date, 10 human and 12 murine TLRs have been identified, and each TLR has a specific set of ligands. Specifically, TLR4 recognizes the lipopolysaccharide (LPS) of gram-negative bacteria^7^,^8^, while TLR3 recognizes viral double-stranded RNA (dsRNA), which is a common intermediate of viral replication and a potent indicator of infection. TLR3 also responds to the synthetic analog polyriboinosinic:polyribocytidylic acid (poly (I:C)) to induce type I interferon (IFN) and inflammatory cytokine/chemokine production^9^,^10^. TLR3 is expressed on immune cells, including myeloid dendritic cells (DCs) and macrophages, as well as non-immune cells such as fibroblasts, epithelial cells, and neurons^11^. Macrophages express TLR3 both on their cell surface and in the early endosome. Although TLR3 on the cell surface participates in dsRNA recognition, TLR3-mediated signaling is initiated from the endosomal compartment^12^,^13^. In addition, RNA released from damaged cells or mRNA can also be recognized by TLR3^14^,^15^. Several studies in TLR3-deficient mice have demonstrated that TLR3 participates in the generation of protective immunity against some viral infections. Numerous studies have documented the importance of TLR cross talk, and multiple signaling pathways contribute to synergistic TLR ligand-dependent cytokine expression. In 2001, Alexopoulou L. *et al*.^16^ reported in *Nature* that following an intraperitoneal injection of LPS into mice, dramatic up-regulation of the expression of TLR3 mRNA was observed in all tissues except the thymus, suggesting that the expression of TLR3 is inducible. Of note, a particularly high expression level of TLR mRNA was observed in lung tissue. These results raised the possibility that bacterial infection can induce sensitivity to viral infection. This observation prompted us to further investigate the mechanism of TLR4-TLR3 cross talk in AMΦ.

In the present study, using both an *in vivo* ALI mouse model and the *in vitro* culture of AMΦ from TLR4^−/−^ and MyD88^−/−^ mice, we demonstrate that LPS up-regulation of TLR3 in AMΦ is dependent on TLR4-MyD88-NF-κB signaling. The functional relevance of the amplification in TLR4-induced TLR3 expression in AMΦ was demonstrated by a marked increase in the expression of the chemokines and cytokines, including macrophage inflammatory protein-2 (MIP-2), macrophage chemoattractant protein-1 (MCP-1), tumor necrosis factor-alpha (TNF-α), and interleukin-6 (IL-6), in the ALI mouse model. Furthermore, we observed alveolar-capillary permeability and the induction of polymorphonuclear leukocyte (PMN) migration in response to sequential challenges with LPS and Poly I:C. Thus, TLR3 up-regulation in AMΦ is dependent on TLR4-MyD88-NF-κB signaling, which may represent an important mechanism responsible for amplifying PMN migration.

## Results

### LPS up-regulates TLR3 expression in a dose- and time-dependent manner in AMΦ

**Figure 1:**
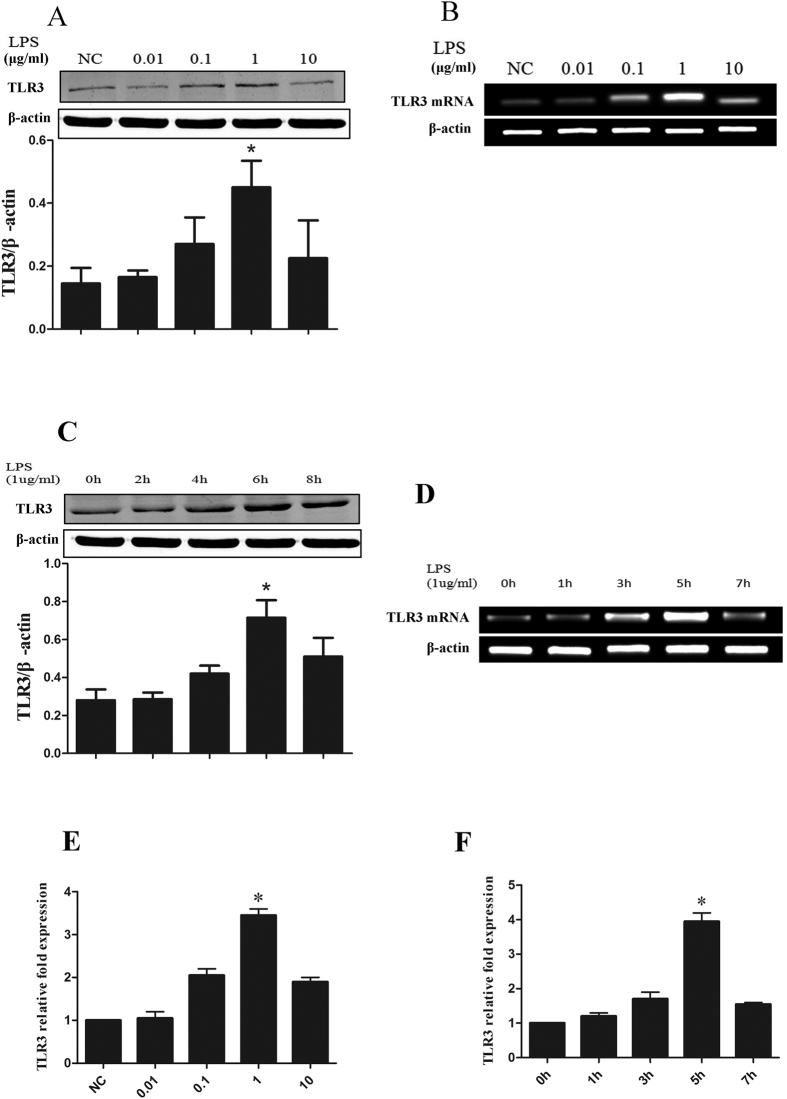
LPS up-regulates TLR3 expression in a dose- and time-dependent manner in alveolar macrophages (AMΦ). **(A**,**B**) Mouse AMΦ were isolated from the BALF of wild-type (WT) mice and stimulated with LPS (0, 0.01, 0.1, 1, or 10 μg/ml) in DMEM containing 10% FBS for 6 h. The graphed values represent the mean ± SEM. Five mice were analyzed per group. (**C**,**D**) Mouse AMΦ were isolated from the BALF of WT mice and stimulated with LPS (1 μg/ml) in DMEM containing 10% FBS for 0–8 h. The graphed values represent the mean ± SEM. Five mice were analyzed per group. (**A,C**) A Western blot of TLR3 protein expression in AMΦ. Actin expression was identified to normalize the densitometry of TLR3 expression. (**B,D**) RT-PCR analysis was used to evaluate TLR3 mRNA expression in AMΦ. β-actin mRNA was detected for normalizing the value of TLR3 mRNA. (**E**,**F**) qRT-PCR analysis was used to evaluate TLR3 mRNA expression in AMΦ. *P < 0.05 compared with the other groups.

To detect the expression of TLR3 in LPS-induced AMΦ, which were isolated from the bronchoalveolar lavage fluid (BALF) of wild-type (WT) mice, LPS was administered at 5 different dosages (0, 0.01, 0.1, 1, and 10 μg/ml). TLR3 expression increased with 0.1 μg/ml but was markedly increased following treatment with 1 μg/ml LPS, and the increase in TLR3 expression trended back to the basal level at 10 μg/ml LPS (Fig. 1A,B). As shown in Fig. 1C, a 1 μg/ml LPS challenge of AMΦ from WT mice was associated with an increase in TLR3 protein expression at 4 h and a more marked increase at 6 h, while the increase in TLR3 expression trended back to the basal level at 8 h. The TLR3 mRNA expression changes shown in Fig. 1D paralleled the protein expression changes. In addition, we obtained the same results using quantitative PCR (Fig. 1E,F). These results suggest that LPS, a TLR4 ligand, can induce TLR3 expression in AMΦ.

### LPS up-regulates TLR3 expression in AMΦ through TLR4-MyD88-NF-κB signaling

**Figure 2:**
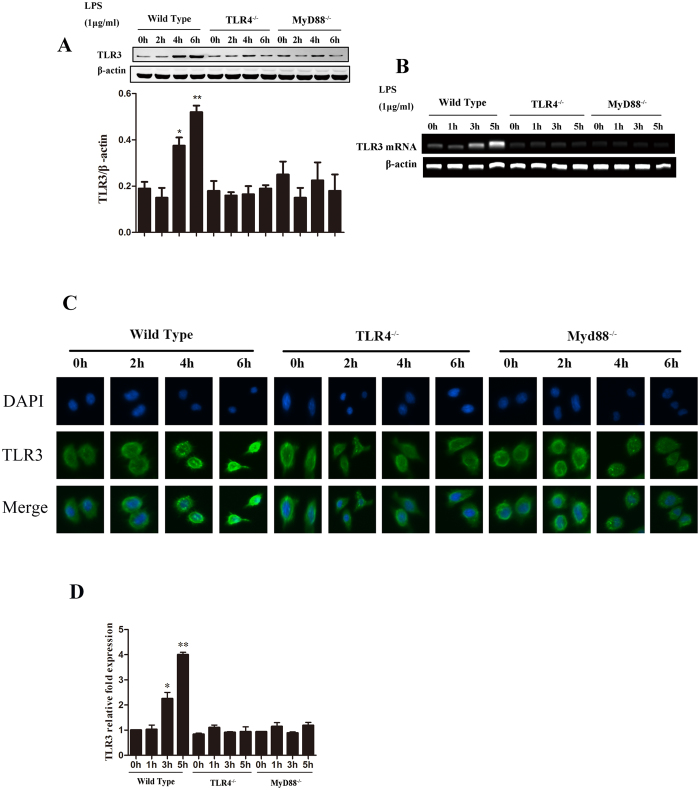
LPS up-regulates TLR3 expression through TLR4-MyD88 signaling in AMΦ. Mouse AMΦ were isolated from the BALF of WT, TLR4^−/−^, and MyD88^−/−^ mice and stimulated with LPS (1 μg/ml) in DMEM containing 10% FBS for 0–6 h. The graphed values represent the mean ± SEM. Five mice were analyzed per group. (**A**) Western blot of TLR3 protein expression in AMΦ. Actin expression was identified to normalize the densitometry of TLR3 expression. (**B**) RT-PCR analysis was used to evaluate TLR3 mRNA expression in AMΦ. β-actin mRNA was detected for normalizing the value of TLR3 mRNA. (**C**) Immunostaining of TLR3 (green). The nuclei are stained with DAPI (blue). The images were acquired using EVOSfl fluorescence microscopy. (**D**) qRT-PCR analysis was used to evaluate TLR3 mRNA expression in AMΦ. *P < 0.05 compared with the other groups; **P < 0.01 compared with the other groups.

As shown in Fig. 2A, LPS (1 μg/ml) challenge of AMΦ, which were isolated from WT mice, resulted in an increase in TLR3 protein expression at 4 h and a marked increase at 6 h. However, in AMΦ from TLR4^−/−^ mice, LPS failed to induce TLR3 expression (Fig. 2A), indicating that TLR4 signaling mediates the LPS-induced up-regulation of TLR3. All TLRs, with the exception of TLR3, utilize MyD88, and TLR4/MyD88 signaling pathway is the primary signaling pathway used to induce the expression of pro-inflammatory cytokines^17^. However, TLR4 can signal through both MyD88-dependent and -independent pathways, and the MyD88-independent signaling for TLR4 results in the production of type I IFNs^18^. To address the role of MyD88 in mediating the LPS-TLR4-induced up-regulation of TLR3, LPS (1 μg/ml) was administered to AMΦ from MyD88^−/−^ mice, and TLR3 expression was assessed. As shown in Fig. 2A, MyD88 deficiency prevented LPS-induced up-regulation of TLR3. The changes in the mRNA expression of TLR3 are shown in Fig. 2B, and these results corresponded to the observed changes in protein expression. As shown in Fig. 2C, the localization of TLR3 was further investigated in AMΦ by immunofluorescence. In the absence of LPS, TLR3 was found within small vesicles throughout the cytoplasm. TLR3-containing vesicles increased in a time-dependent manner in AMΦ from WT mice, but not in TLR4^−/−^ or Myd88^−/−^ mice, and similar results were observed using quantitative PCR (Fig. 2D).

### NF-κB mediates LPS-TLR4-induced up-regulation of TLR3

**Figure 3:**
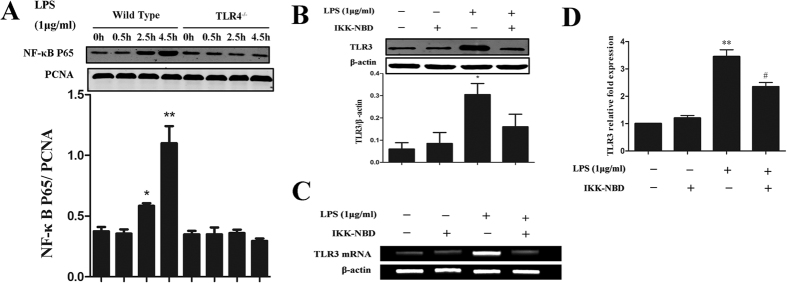
NF-κB mediates LPS-TLR4 signaling-induced TLR3 up-regulation in AMΦ. Mouse AMΦ were isolated from the BALF of WT and TLR4^−/−^ mice and stimulated with LPS (1 μg/ml) in DMEM containing 10% FBS. The graphed values represent the mean ± SEM. Five mice were analyzed per group. (**A**) Western blot of NF-κB P65 protein expression in the nuclear fraction of AMΦ. PCNA expression was identified to normalize the densitometry of NF-κB p65 expression. (**B**,**C**) AMΦ were treated with LPS (1 μg/ml) in the presence or absence of the NF-κB inhibitor IKK-NBD (100 μM), and then TLR3 protein expression was detected by Western blotting (**B**) and TLR3 mRNA expression was detected using RT-PCR (**C**). qRT-PCR analysis was used to detect TLR3 mRNA expression in AMΦ (**D**). *P < 0.05 compared with the other groups; **P < 0.01 compared with the other groups.

Activation of both NF-κB and MAPK can occur in the presence of MyD88. NF-κB p65 was detected using nuclear protein extracts from AMΦ to determine whether LPS-TLR4 up-regulation of TLR3 was the result of activation of NF-κB. As shown in Fig. 3A, in response to LPS in WT mice, the expression of NF-κB p65 increased at 2.5 h and reached a significant level at 4.5 h. However, in TLR4^−/−^ mice, LPS failed to induce nuclear NF-κB p65 expression.

To address the role of NF-κB in mediating the LPS-induced up-regulation of TLR3, we determined the effects of IKK-NBD, an NF-κB inhibitor^19^,^20^, on LPS-induced TLR3 expression in AMΦ. As shown in Fig. 3B,C, IKK-NBD (100 μM) prevented the LPS-induced up-regulation of TLR3 mRNA and protein expression in AMΦ isolated from WT mice. We observed similar results by quantitative PCR (Fig. 3D). These results demonstrate that NF-κB signaling plays a role in mediating TLR4-TLR3 cross talk.

### Increased TLR3 expression results in enhanced MIP-2 expression and PMN migration

**Figure 4:**
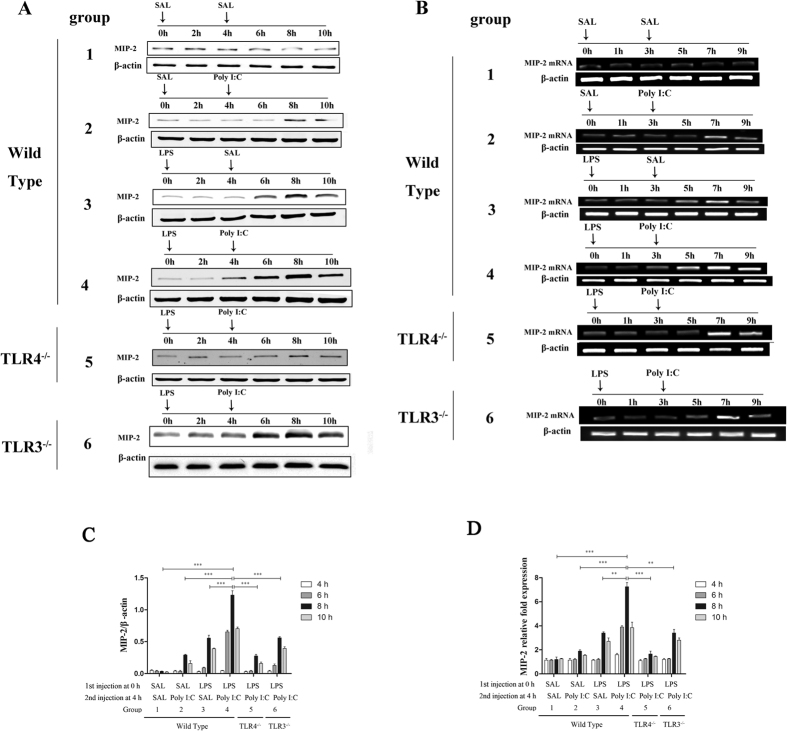
Increased TLR3 expression results in augmented MIP-2 expression in the lung. The results show the effects of sequential challenges with LPS and Poly I:C on MIP-2 expression in the lungs. In WT mice, LPS (5 μg/g) or saline (SAL) was injected intratracheally at time 0 h, and 4 hours later, Poly I:C (5 μg/g) or SAL was injected. In TLR4^−/−^ and TLR3^−/−^ mice, LPS was injected intratracheally (5 μg/g) at time 0 h, and Poly I:C (5 μg/g) was injected intratracheally at 4 h. Lung tissue was harvested at the indicated times, and MIP-2 protein expression was detected using Western blotting (**A**,**C**), with actin being used to normalize the densitometry of MIP-2 expression. Additionally, MIP-2 mRNA expression was detected using RT-PCR (**B**), and qRT-PCR was used to detect MIP-2 mRNA expression in AMΦ (**D**). β-actin mRNA was used to normalize the value of MIP-2 mRNA expression. The graphed values represent the mean ± SEM. Five mice were analyzed per group. **P < 0.01 compared with the other groups; ***P < 0.001 compared with the other groups.

To address the physiological relevance of LPS/TLR4 activation and TLR3 expression in AMΦ, we assessed MIP-2 expression in the lungs using sequential challenges of LPS and Poly I:C. Injection of LPS at time 0 followed by a saline injection (Group 3) at 4 h resulted in a marked increase in MIP-2 expression by 6 h after LPS challenge, which was further increased at 8 h and then returned to the basal level by 10 h after LPS challenge (Fig. 4A). Saline injection at time 0 and Poly I:C injection (5 μg/g body weight, intratracheally administered) (Group 2) at 4 h caused a very small increase in MIP-2 expression. However, the sequential injection of LPS at time 0 and Poly I:C injection at 4 h (at a time when TLR3 was up-regulated) (Group 4) stimulated augmented MIP-2 expression compared with single LPS (Group 3) or Poly I:C (Group 2) challenge (Fig. 4A). In contrast, the sequential injection of Poly I:C at 4 h after LPS failed to induce increased MIP-2 expression in TLR4^−/−^ mice (Group 5) compared with single Poly I:C challenge (Group 2) (Fig. 4A), and sequential challenge with Poly I:C after LPS caused only a similar increase as that observed for LPS alone in TLR3^−/−^ mice (Group 6). The changes in the mRNA expression of MIP-2 shown in Fig. 4B paralleled those observed for protein expression.

**Figure 5:**
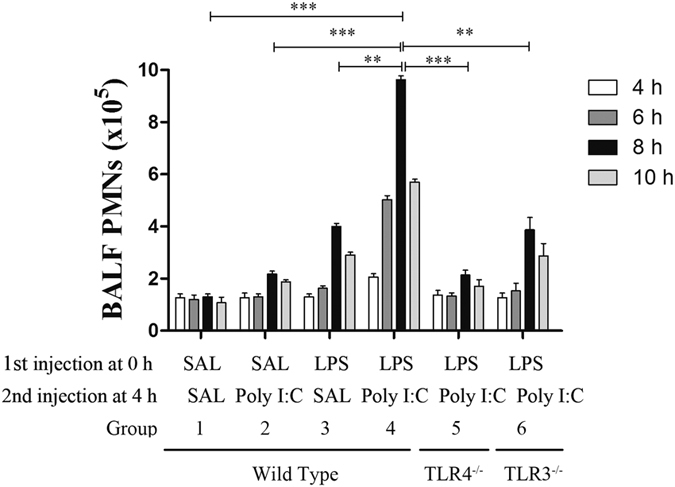
Increased TLR3 expression results in PMN migration into the airspaces. The results show the effects of sequential challenges with LPS and Poly I:C on PMN migration into the airspaces. In WT mice, LPS (5 μg/g) or saline (SAL) was injected intratracheally at time 0 h, and 4 h later, Poly I:C (5 μg/g) or SAL was injected. In TLR4^−/−^ and TLR3^−/−^ mice, LPS (5 μg/g) was injected at time 0 h, and Poly I:C (5 μg/g) was injected intratracheally at 4 h. PMN numbers in BALF were counted at the times indicated. The graphed values represent the mean ± SEM. Five mice were analyzed per group. **P < 0.01; ***P < 0.001 compared between the groups as indicated.

Because AMΦ production of chemokines, such as MIP-2, is an important determinant of PMN migration, we also addressed the role of LPS/TLR4-mediated up-regulation of TLR3 in regulating PMN migration in the lungs. PMNs were counted in the BALF of WT, TLR4^−/−^, and TLR3^−/−^ mice. In the LPS (Group 3) and Poly I:C (Group 2) only injection groups, MIP-2 caused a slight increase in PMN migration in WT mice when compared with the saline control (Group 1). Sequential injection of Poly I:C at 4 h after the initial LPS injection (Group 4) markedly increased PMN migration in WT mice compared with the other groups. However, sequential challenge with LPS and Poly I:C did not significantly increase PMN migration in TLR4^−/−^ mice compared with WT mice (Fig. 5). Moreover, there was a similar increase in AMΦ after sequential challenge with LPS and Poly I:C in TLR3^−/−^ mice (Group 6) when compared to LPS alone (Group 3). Additionally, the observed MIP-2 expression level in the lung was consistent with PMN migration. Together, these data show the important role of TLR4 signaling and AMΦ MIP-2 expression in mediating PMN migration in response to the up-regulation of TLR3 expression.

### Increased TLR3 expression in AMΦ results in enhanced cytokine expression and ALI

**Figure 6:**
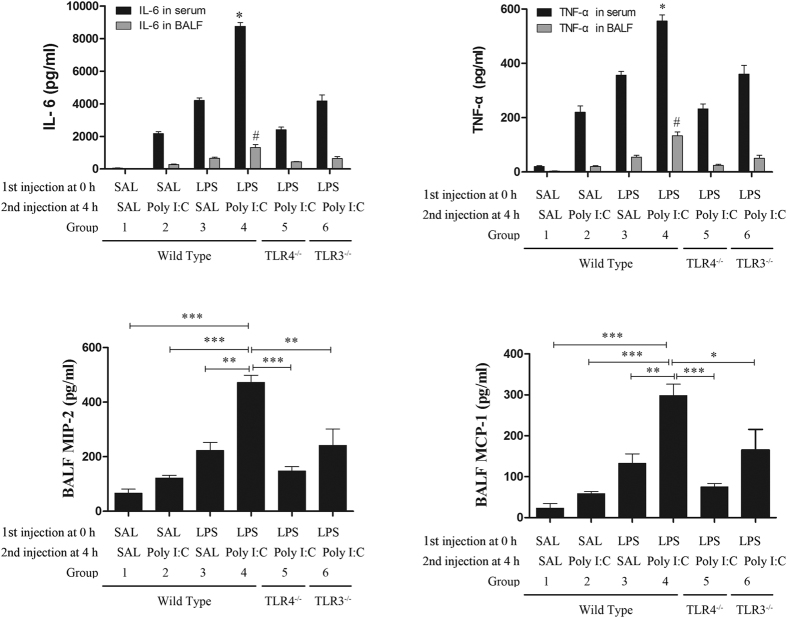
Increased TLR3 expression results in enhanced cytokine and chemokine expression in the serum and BALF. The results show the effects of sequential challenges with LPS and Poly I:C on cytokine and chemokine expression in the serum and BALF. In WT mice, LPS (5 μg/g) or saline (SAL) was injected intratracheally at time 0 h, and 4 h later, Poly I:C (5 μg/g) or SAL was injected. In TLR4^−/−^ and TLR3^−/−^ mice, LPS (5 μg/g) was injected at 0 h, and Poly I:C (5 μg/g) was injected intratracheally at 4 h. The serum and BALF were collected at 8 h, and the cytokines and chemokines were detected using ELISA. (**A**) IL-6 in the serum and BALF; (**B**) TNF-α in the serum and BALF; (**C**) MIP-2 in the BALF; (**D**) MCP-1 in the BALF. The graphed values represent the mean ± SEM. Five mice were analyzed per group. *P < 0.05 compared with the other groups in the serum; *P* < 0.05 compared with the other groups in the BALF; *P < 0.05; **P < 0.01; ***P < 0.001 compared between the groups as indicated; ^#^*P* < 0.05 compared between the groups as indicated.

To address the effect of LPS/TLR4-mediated activation of TLR3 in AMΦ on inflammatory cytokines, we assessed TNF-α and IL-6 in the serum and BALF, as well as the chemokines MIP-2 and MCP-1 in the BALF, following sequential intratracheal challenges with LPS and Poly I:C. In the ALI animal model, LPS was injected intratracheally at time 0, and Poly I:C was injected intratracheally at 4 h (the time point when TLR3 was up-regulated in AMΦ as described above). Enzyme-linked immunosorbent assays (ELISAs) were used to assess MIP-2 and MCP-1 in the BALF as well as IL-6 and TNF-α in both the BALF and serum at 8 h. LPS (Group 3) or Poly I:C (Group 2) alone induced a slight increase in MIP-2, MCP-1, IL-6, and TNF-α when compared with the saline control (Group 1) (Fig. 6), whereas sequential challenge with LPS and Poly I:C (Group 4) stimulated a marked increase in MIP-2, MCP-1, IL-6, and TNF-α expression. However, the sequential challenge with LPS and Poly I:C did not significantly increase MIP-2, MCP-1, IL-6, and TNF-α in TLR4^−/−^ mice (Group 5) when compared to WT mice (Group 4). The sequential challenge with LPS and Poly I:C also led to a similar increase in TLR3^−/−^ mice (Group 6) when compared to the LPS alone group (Group 3) (Fig. 6).

**Figure 7:**
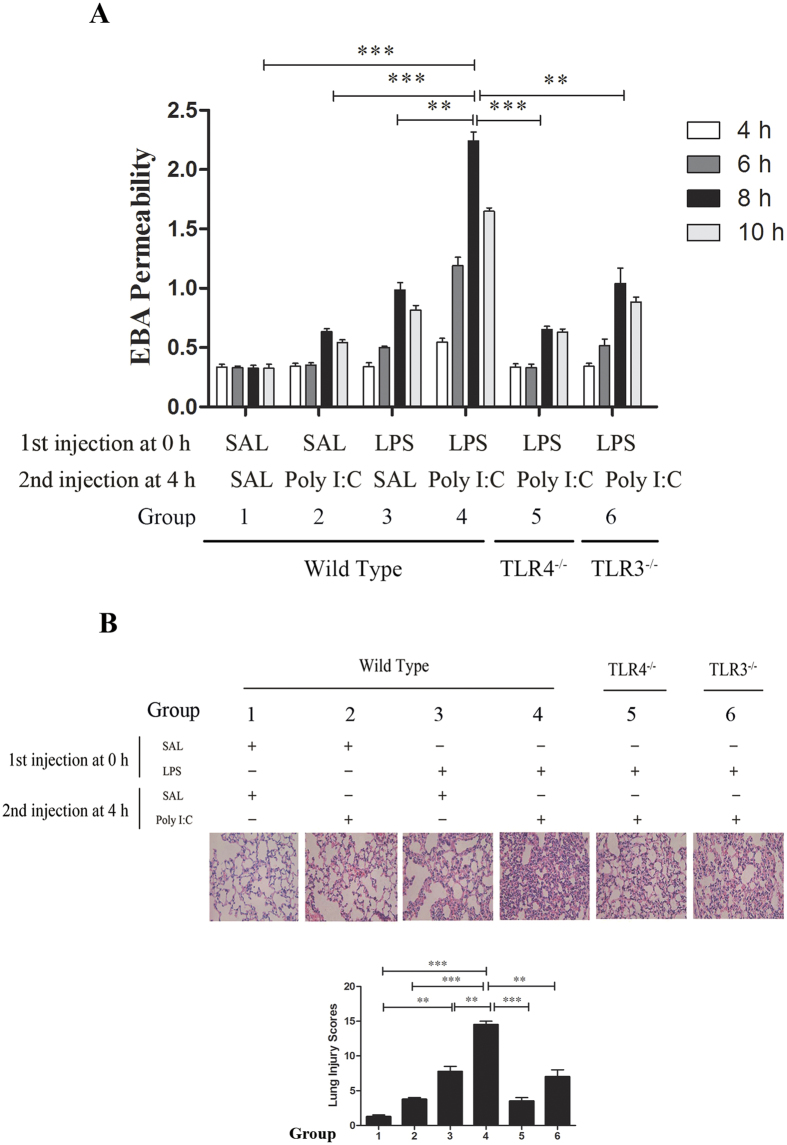
Increased TLR3 expression results in enhanced ALI. The results show the effects of sequential challenges with LPS and Poly I:C on alveolar-capillary permeability and histological changes in the lung. In WT mice, LPS (5 μg/g) or saline (SAL) was injected intratracheally at time 0 h, and 4 h later, Poly I:C (5 μg/g) or SAL was injected. In TLR4^−/−^ and TLR3^−/−^ mice, LPS (5 μg/g) was injected at 0 h, and Poly I:C (5 μg/g) was injected intratracheally at 4 h. Lung tissue and serum were harvested at the times indicated, and alveolar-capillary permeability was detected by using Evan’s blue staining (**A**). Lung tissue was collected at the time indicated and subjected to hematoxylin and eosin (H&E) staining (x200 magnification) (**B**). **P < 0.01; ***P < 0.001 compared between the groups as indicated.

Because chemokine-dependent PMN migration and inflammatory cytokine secretion are important determinants of ALI, we next addressed the role of LPS/TLR4-mediated up-regulation of TLR3 in alveolar-capillary permeability using Evan’s blue, which binds albumin, in WT, TLR4^−/−^, and TLR3^−/−^ mice. We assessed permeability at 4, 6, 8, and 10 h after LPS/Poly I:C administration. As shown in Fig. 7A, at the 8 h time point, either LPS (Group 3) or Poly I:C (Group 2) alone caused a slight increase in permeability in WT mice when compared to the saline (SAL) control (Group 1). In contrast, LPS/Poly I:C (Group 4) caused a further increase in alveolar-capillary permeability in WT mice when compared with the LPS/SAL group. In TLR4^−/−^ mice (Group 5), alveolar-capillary permeability was markedly attenuated in response to LPS/Poly I:C challenge compared with WT mice (Group 4). The alveolar-capillary permeability was similar to the results observed for LPS alone (Group 3) when TLR3^−/−^ mice were used (Group 6). As shown in Fig. 7B, the histopathology of lung tissues was also assessed 8 h following LPS/Poly I:C administration. Injection of LPS at time 0 followed by a saline injection (Group 3) at 4 h caused marked changes in lung histopathology at 8 h. A saline injection at time 0 and a Poly I:C injection (5 μg/g body weight, intratracheally administered) at 4 h resulted in minimal changes (Group 2). However, the sequential injection of LPS at time 0 and Poly I:C injection at 4 h (at a time when TLR3 was up-regulated) in WT mice (Group 4) led to augmented changes in histopathology, including diffuse interstitial edema and inflammatory cell infiltration, compared with the single LPS or Poly I:C challenge. In contrast, the sequential injection of Poly I:C at 4 h after LPS failed to induce augmented histopathology changes in TLR4^−/−^ mice (Group 5) compared with WT mice. In addition, we did not observe augmented histopathology changes in TLR3 mice (Group 6) and instead observed a change similar to that observed for LPS alone (Group 3). Taken together, these data show the important role of LPS/TLR4 signaling in the up-regulation of TLR3 expression in ALI.

## Discussion

AMΦ, which are located in the alveolar compartment of the lungs, provide a key initiation signal for ALI^21^,^22^,^23^. Previous studies have shown that the later phase of ALI is neutrophil-dependent^24^, while AMΦ contribute to the acute phase of lung injury. Once activated by bacteria or viruses, AMΦ generate and release a multitude of mediators, such as cytokines (IL-6, TNF-α) and chemokines (MCP-1, MIP-2)^25^,^26^. These mediators act as chemoattractants for the migration of large numbers of activated inflammatory cells, such as monocytes and neutrophils, into the airspaces^27^.

Synergy between viral and bacterial TLR signaling, which leads to amplification of the inflammatory response, has been reported previously^28^. Additionally, Tian X. *et al*. found that Poly I:C enhanced susceptibility to secondary pulmonary infections by bacteria^29^. Co-stimulation with LPS and Poly I:C markedly enhances the immune response, although the mechanism for this combined effect remains poorly understood. Alexopoulou L. *et al*.^16^ found that when LPS was injected intraperitoneally, TLR3 expression in lung tissue was dramatically up-regulated, which suggests that the expression of TLR3 is inducible. Pan *et al*.^30^ provided direct evidence that LPS induces TLR3 expression via a TLR4-MyD88-IRAK-TRAF6-NF-κB-dependent signaling pathway in human peripheral blood monocytes and monocytic cell lines, such as THP-1 cells. Furthermore, we observed that LPS could induce TLR3 expression in a dose- and time-dependent manner in AMΦ. The stimulation of TLR4 by LPS induces the release of critical proinflammatory cytokines that are necessary to activate a potent immune response. Indeed, LPS/TLR4 signaling has been intensively studied in recent years^31^,^32^,^33^. In our study, the role of TLR4 signaling in regulating TLR3 expression was clearly shown using TLR4^−/−^ mice. LPS challenge in WT mice induced the up-regulation of TLR3, whereas this response was impaired in TLR4^−/−^ mice. The major adaptor molecules that bind to the intracellular domain of TLR4 are MyD88 and TRIF^34^,^35^, and our results showed that MyD88 mediated the TLR4-TLR3 cross talk, as LPS challenge of MyD88^−/−^ mice failed to induce TLR3 expression. Furthermore, the activation of NF-κB was associated with an increase in TLR3 expression after LPS challenge and a reduced expression of TLR3 in AMΦ in which NF-κB was inhibited by IKK-NBD. Therefore, these results demonstrate an important role for TLR4-MyD88-NF-κB in mediating TLR3 expression.

**Figure 8:**
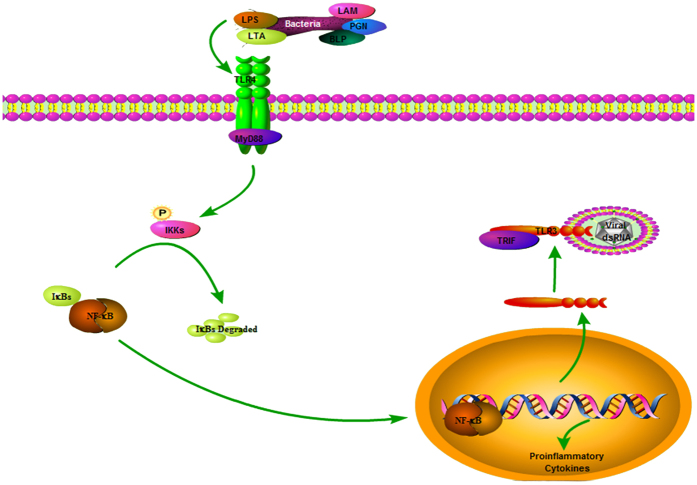
A model demonstrating how LPS/TLR4/MyD88-NF-κB signaling regulates TLR3 expression in AMΦ. LPS-induced TLR3 expression in AMΦ is possibly mediated by TLR4-MyD88-NF-κB signaling. An increase in TLR3 expression results in an amplified response to Poly I:C, which augments cytokine and chemokine expression and enhances PMN migration. Finally, TLR4-TLR3 cross talk leads to ALI.

ALI is caused by an uncontrolled systemic inflammatory response that results from direct (aspiration, pneumonia, ventilation-induced lung injury, etc.) or indirect injury (sepsis, hemorrhagic shock, etc.), leading to the activation of AMΦ and the sequestration of neutrophils^3^. Excessive recruitment of leukocytes is critical to the pathogenesis of ALI. Neutrophils are the first leukocytes to be recruited to sites of inflammation in response to chemokines released by activated AMΦ^36^. Specifically, stimulation of AMΦ leads to the release of chemokines, which induce neutrophils to migrate from the intravascular space across the endothelium and epithelium into the airspaces^27^. According to the relative position of the cysteine residues, chemokines have been classified into four subfamilies (CXC, CC, C, and CX3C). Among these, MIP-2, which is also known as CXCL2, is thought to play a major role in mediating neutrophil recruitment^37^. Belperio J.A. *et al*.^38^ found that lung expression of MIP-2 was correlated with lung injury and neutrophil sequestration during the pathogenesis of ventilation-induced lung injury, and CXCR2^−/−^ mice show a marked reduction in neutrophil sequestration and lung injury. Additionally, MIP-2 was shown to be up-regulated in the lungs and BALF of animals, which was associated with neutrophil accumulation in the lungs after LPS administration^39^,^40^,^41^. Furthermore, Villar J. *et al*. showed that a CXCL2 polymorphism is associated with better outcomes in patients with severe sepsis^42^. In the present study, we observed that LPS/TLR4 signaling up-regulated TLR3 expression in AMΦ. This cross talk between TLR4 and TLR3 in AMΦ resulted in the amplification of cytokine (IL-6, TNF-α) and chemokine (MIP-2, MCP-1) expression in response to LPS and Poly I:C, which activate TLR4 and TLR3, respectively, and subsequently led to enhanced PMN sequestration into the lung, which was found to be correlated with ALI based on the assessment of alveolar-capillary permeability and histological sections of lung tissue. Thus, the present study demonstrates a novel mechanism by which LPS can induce AMΦ to up-regulate TLR3 expression, through a TLR4-Myd88-NF-κB-dependent pathway, thereby sensitizing AMΦ to TLR3 ligands and promoting enhanced lung inflammation (Fig. 8).

In clinical scenarios, a variety of pathogens are involved in lung infections. In particular, viral infections are a significant risk factor for acquiring bacterial infections^43^,^44^. TLR3 is thought to be a major mediator of the cellular response to viral infection, because it responds to dsRNA, a common by-product of viral replication^45^. TLR3 has been implicated in infections by mouse cytomegalovirus (MCMV), reoviruses, lymphocytic choriomeningitis virus (LCMV), and influenza A virus (IAV)^9^. TLR3^−/−^ mice are more resistant to lethal West Nile virus, a mosquito-borne ssRNA flavivirus, which causes human disease of variable severity^46^, and show reduced inflammation and lethality upon IAV infection^47^,^48^. Thus, up-regulation of TLR3 may contribute to an increased lung immune response to viral infection following a bacterial infection. Despite the relatively well-known role of viral infections in promoting bacterial infections, it is still not clear whether bacterial infections also promote viral infections. Therefore, our results are the first to offer new insight into this topic. There is also a growing body of evidence showing that most patients have a mixture of bacterial and viral infection^49^,^50^, and the cross talk between TLR4 and TLR3 induces a synergistic inflammatory response in cases of mixed infection. Cameron R.J. *et al*.^49^ found that bacterial and viral pathogens interact to cause additional increases in inflammatory markers and an exacerbation of disease severity in patients with chronic obstructive pulmonary disease (COPD). TLR3 has been shown to respond to dsRNA, a replication intermediary for many viruses, but the heterologous RNA released from necrotic cells or that is generated by *in vitro* transcription, such as mRNA, also stimulates TLR3 signaling and induces immune activation. Indeed, Cavassani KA^15^ observed TLR3 activation during experimental polymicrobial sepsis and ischemia gut injury in the absence of an exogenous viral stimulus, and TLR3^−/−^ mice were protected from the lethal effects of sustained inflammation. Moreover, treatment with a TLR3 antibody could attenuate the tissue injury associated with gut ischemia and significantly decrease sepsis-induced mortality. Therefore, TLR3 serves as a regulator of the amplification of the immune response as well as an endogenous sensor of necrosis, independent of viral activation. Our study showed that up-regulation of TLR3 significantly amplified IL-6, TNF-α, MCP-1, and MIP-2 expression, which then enhanced PMN migration and finally led to ALI.

TLR cross talk has obvious advantages for the host in protecting against infectious agents, because it enhances the initial immune reaction to pathogen infection and better primes the host for mounting a more robust adaptive immune response. The concept that multiple TLR-ligand interactions are required for effective host resistance to pathogens has important implications for the design of vaccinations and immunotherapies against infectious diseases. Several studies have convincingly shown the improved efficacy of treatment with multiple TLR ligands compared with single TLR ligands in stimulating cellular immune responses *in vivo*. For example, co-administration of poly I:C and CpG ODNs increased serum cytokine production in a mouse tumor model when compared to the administration of either of the ligands alone^51^. Bone-marrow-derived DCs exposed to both poly I:C and a TLR7 ligand more effectively stimulated cytotoxic T lymphocyte responses compared to DCs exposed to either TLR ligand alone^52^. These studies provide an important conceptual foundation to examine the protective efficacy of multiple TLR-ligand combinations in vaccines against infectious diseases. However, synergistic amplification of the inflammation response may be detrimental, because it can result in immune over-activation, which hampers immune homeostasis. As a consequence of an overactive response, the function of various organ systems may be compromised, resulting in multiple organ dysfunction syndrome (MODS) and death. Cytokines (TNF-α and IL-6) are important components of the immune system because they act as messengers between cells, but they are also involved in many pathological aspects of the cascade leading to systemic inflammatory response syndrome (SIRS) and ultimately MODS. According to the two-hit hypothesis^53^, patients who survive the initial inflammatory insult may die following a relatively minor second event that would not normally be life-threatening. In this study, viral (poly I:C) stimulation as a relatively minor secondary insult, led to an exaggerated secondary inflammatory response. Thus, understanding the mechanism of this two-hit phenomenon may help to devise novel therapeutic strategies to prevent overwhelming and life-threatening inflammatory conditions such as septic shock and trauma-induced SIRS.

## Materials and Methods

### Animals

Male C57BL/6 wild type mice were purchased from the Laboratory Animal Research Center of Shanghai. TLR4 knockout (TLR4^−/−^) mice, MyD88 knockout (MyD88^−/−^) mice and TLR3 knockout (TLR3^−/−^) were obtained from Dr. Billiar’s lab at the University of Pittsburgh. All mice used are on a C57BL/6 background. All experimental protocols involving animals were approved by Institution Animals Care and Use Committee of Tongji and Pittsburgh University. The experiments were performed in accordance with the National Institutes of Health Guidelines for the Use of Laboratory Animals. Mice were 8–12 weeks of age at the time of experiments and were maintained on standard rodent chow and water *ad libitum*. Animals were anesthetized with 100 mg/kg ketamine and 10 mg/kg xylazine administered intraperitoneally. Animals were intratracheally administered LPS (5 μg/g body wt; *Escherichia coli* O111:B4; Sigma, St. Louis, MO) in 50 μl of saline (SAL) or SAL at first time, then after 4 h intratracheally administered Poly I:C (5 μg/g body wt; 31852-29-6; Invivogen) in 50 μl of saline (SAL) or SAL alone at the second time using a MicroSpray syringe. The animals were randomly in one of four groups: SAL/SAL, SAL/Poly I:C, LPS/SAL, and LPS/Poly I:C. At various time points, a 20G sterile catheter was inserted into the trachea to collect bronchoalveolar lavage fluid (BALF) as previously described^54^. Blood samples were immediately obtained by cardiac puncture and transferred to the laboratory for analysis of cytokines (IL-6, TNF-α), chemokines (MIP-2, MCP-1) in serum and BALF by ELISA. PMN counts in BALF were determined on Wright-Giemsa-stained slides. Briefly, total cell counts were determined on a grid hemocytometer. Then a total of 500 cells were counted in cross-section per sample, and the number of PMNs was calculated as the total cell count times the percentage in the BALF sample. The lungs were rapidly removed from all mice and washed in ice-cold saline. Half of the lung tissues were stored at −80 °C prior to biochemical analyses including MIP-2 expression in lung lysates was measured by Western blotting and RT-PCR, while the other half of the lung tissue was fixed in 4% formalin solution in preparation for histopathological analyses. For histological analysis, lung tissue samples were fixed in 4% paraformaldehyde in PBS overnight at 4 °C. The samples were then dehydrated, embedded in paraffin, and cut into 5 μm sections. After deparaffinization, the tissues were stained with hematoxylin and eosin (H&E) for histological analysis. Lung sections were scored for lung injury, including the following: (1) alveolar and capillary edema, (2) intravascular and peri-bronchial influx of inflammatory cells, (3) thickness of the alveolar wall, and (4) hemorrhage. The items were semiquantitatively scored as none, minimal, light, moderate, or severe (score 0, 1, 2, 3 or 4, respectively) by a pathologist blinded to the experimental group. The lung injury score was obtained by averaging the score from the animals within each group.

### AMΦ isolation

BAL was performed as previously described^54^. Normally, the BAL fluid contains ~91% of AMΦ and ~9% of other cells, including PMN and lymphocytes. The immunomagnetic separation system was used to isolate AMΦ. Magnetic nanoparticle-conjugated antibody (CD11b MicroBeads, Miltenyi Biotec) was chosen to label and remove PMN and lymphocytes. The resulting cells consisted of >98% macrophages, and cell viability was >95%. AMΦ from wild type, TLR4^−/−^, TLR3^−/−^ and MyD88^−/−^ mice were cultured in DMEM containing 10% FBS and 10 μl/ml penicillin/streptomycin for 2 days, then they were washed three times with PBS and the medium was changed to low-serum medium (1% FBS). Cells were stimulated with LPS (NC, 0.01, 0.1, 1, 10 μg/ml) for 0–8 h in DMEM containing 10% FBS at a concentration of 1 × 10^6 ^cells/ml of medium. TLR3 expression in the AMΦ lysates was measured by Western blotting and RT-PCR.

### Measurement of alveolar-capillary permeability

Alveolar-capillary permeability was assessed with Evans blue albumin (EBA) as previous described^54^,^55^. Evans blue (0.5% EB, Sigma-Aldrich, St Louis, MO, USA) was dissolved in Ca^2+^/Mg^2+^-free phosphate-buffered saline (PBS; Sigma-Aldrich), and conjugated to albumin (4% EBA) that was prepared by adding bovine serum albumin (Sigma-Aldrich). After thoroughly dissolving by gently stirring with a magnetic bar, the EBA solution was filtered through a 0.22 μm syringe filter and aliquots were stored at −80 °C until use. Each aliquot was used only once for each experiment. To evaluate alveolar-capillary barrier function, EBA (25 mg/kg body weight) was injected into the internal jugular vein 1 h before euthanasia and lung harvesting. Blood samples were obtained from the right heart, and the pulmonary vasculature was subsequently infused with 1 mL PBS. The right lung was ligated at the level of the right mainstem bronchus, excised, blotted dry, weighed and stored in liquid nitrogen until these samples were used for EBA analysis. After freeze/thaw, the lung tissue was homogenized in 2 mL PBS and incubated with an additional 2 mL of formamide (Sigma-Aldrich) (18 h; 60 °C). Formamide extracts were centrifuged (15,000 g × 30 min; 4 °C), and the centrifuged supernatants were collected to quantify lung EBA content using a dual-wavelength (620 nm and 740 nm) spectrophotometric method. Pulmonary EBA absorbance at 620 nm was corrected by a correction factor with EBA absorbance at 740 nm. The EBA permeability index was calculated by dividing pulmonary EBA absorbance at 620 nm/g of lung tissue by plasma EBA absorbance at 620 nm.

### Nuclear protein extraction

Nuclear protein extracts were prepared from AMΦ following the kit instructions of Thermo Scientific (NE-PER Nuclear and Cytoplasmic Extraction Reagents, 78833, Thermo Fisher). AMΦ were harvested with trypsin-EDTA and then centrifuge at 500 × g for 5 minutes. After washing cells twice with PBS, pellet by centrifugation at 500 × g for 3 minutes and discarded the supernatant. Adding CER I (protease inhibitors and PMSF) to the cell pellet, vortex the tube vigorously for 15 s, then incubated the tube on ice for 10 minutes. Added CER II to the tube and vortex for 5 s on the highest setting, incubated the tube on ice for 1 minute. The tube was centrifuged for 5 minutes at 16,000 × g, then transferred the supernatant (cytoplasmic extract) to a clean pre-chilled tube. Nuclei pellet was suspended in NER and vortex for 15 s. Placed the sample on ice and continued vortexing for 15 s every 10 minutes, for a total of 40 minutes. After centrifuged at 16,000 × g for 10 minutes, supernatants containing nuclear proteins were frozen in liquid nitrogen in small aliquots and store at −80 °C. Protein quantification was performed using BCA protein assay.

### Western blot analysis

AMΦ and lung tissues were lysated in radio-immunoprecipitation lysis buffer (RIPA), protease inhibitors (Roche, Mannheim, Germany) and phenylmethylsulfonyl fluoride (PMSF). Protein concentrations were subsequently determined by standard BCA assay. After addition of 6 × sodium dodecyl sulfate (SDS) loading buffer, equivalent amounts of protein were heated (100 °C; 5 min) and separated by gel electrophoresis using a 10% SDS-polyacrylamide electrophoresis gel. Resolved proteins were then transferred to a nitrocellulose membrane and blocked with Tris-buffered saline containing Tween-20 (TBST) and 5% nonfat milk (1 h; 24 °C). Nitrocellulose membranes were incubated overnight at 4 °C with primary antibody against TLR3 (ab62566; Abcam, Hong Kong, China), NF-κB p65 (ab7970, Abcam, Hong Kong, China), Abcam, MIP-2 (ab25130, Hong Kong, China), PCNA (ab18197, Abcam, Hong Kong, China) and β-actin (ab8226, Abcam, Hong Kong, China). The membranes were washed in TBST three times, incubated with secondary antibody (926-32221 IRDye 680 mouse-anti-rabbit secondary antibody; Licor Biosciences, Lincoln, NE, USA) for 1 h at 37 °C and then washed in TBST three additional times. The membranes were determined by using an Odyssey image analysis system (Licor Biosciences). Western blots were quantitated using Quantity One software (Bio-Rad, Foster City, CA, USA) and normalized to β-actin and PCNA signal.

### Reverse transcription PCR (RT-PCR)

Total RNA was extracted from AMΦ and lung tissues using the TRIzol reagent (Sigma-Aldrich) and following the manufacturer’s instructions. Total RNA was then reverse transcribed using a PrimeScript RT reagent kit (TaKaRa Bio Inc. Shiga, Japan). Primers for TLR3 amplification (162 bp) were: position 91 forward 5′-GTGAGATACAACGTAGCTGACTG-3′, position 231 reverse 5′-TCCTGCATCCAAGATAGCAAGT-3′. Primers for MIP-2 amplification (108 bp) were: position 62 forward 5′-CCAACCACCAGGCTACAGG-3′, position 169 reverse 5′-GCGTCACACTCAAGCTCTG-3′. Primers for β-actin amplification (154 bp) were: position 163 forward 5′-GGCTGTATTCCCCTCCATCG-3′, position 295 reverse 5′-CCAGTTGGTAACAATGCCATGT-3′. The product of reverse transcription was amplified by following the Premix Taq Version 2.0 instructions (TaKaRa Bio Inc.). PCR products were separated using a 2% agarose gel and identified by SYBR green staining. Expression of mRNA was quantitated using Image Lab software (Bio-Rad) and normalized to the β-actin signal.

### Quantitative real-time PCR

Quantitative real-time PCR (qPCR) reactions were performed using Fast SYBR Green Master Mix (Thermo Fisher) in Applied Biosystems 7900HT Fast Real-Time PCR system according to the manufacturer’s instructions. The cycling conditions were 95 °C for 10 min followed by 40 cycles of 95 °C for 10 s, 60 °C for 30 s. At the end of the last cycle, the temperature was increased from 65 °C to 95 °C (0.1 °C/s) to produce a melting curve. The specificity of amplification was assessed for each sample by melting curve analysis. Each PCR product showed a single peak. The size of the amplicon was checked by electrophoresis. Agarose gel electrophoresis revealed a single product of the expected size. The analysis of the expression of TLR3 relative to the β-actin was performed with software in relative quantification mode following the manufacturer’s instruction. The following criteria of sequences of all primers used in this study were applied in the course of designing the primers: product size from 100 to 500 bp, primer size from 17 to 23 bp, and a mean melting temperature of 60 °C.

### Immunofluorescence staining of cells and florescence microscopy

AMΦ were cultured for a defined time period, fixed in 4% paraformaldehye/1 × PBS for 15 min. Cells were washed three timers with 1 × PBS and permeabilized using 0.1% Triton X-100 in 1 × PBS, and blocked with 5% BSA for 45 min and sequentially administered primary antibody and secondary antibody (Alexa-488-conjugated donkey anti rabbit secondary antibody). Nuclei were stained with DAPI for immunofluoresence analysis. Stained cells were examined and recorded using EVOSfl fluorescence microscopy.

### Statistics

The Data are presented as the means ± SEM of the indicated number of experiments and analyzed using ANOVA; post hoc testing was performed using the Bonferroni modification of the *t*-test. The individual studies performed throughout this work represent at least five independent studies. Power analyses were performed by using a Type I error probability of 0.05, with a power of 0.9, to determine the sample size necessary to reject the null hypothesis. All statistical analyses were carried out using the GraphPad Prism 5 program.

## Additional Information

**How to cite this article**: Ding, X. *et al*. TLR4 signaling induces TLR3 up-regulation in alveolar macrophages during acute lung injury. *Sci. Rep.* **7**, 34278; doi: 10.1038/srep34278 (2017).

**Publisher's note:** Springer Nature remains neutral with regard to jurisdictional claims in published maps and institutional affiliations.
